# Associations Between Actigraphy-Assessed Health Behaviors and Cognitive Performance in Older Adults

**DOI:** 10.1093/geroni/igab046.153

**Published:** 2021-12-17

**Authors:** Alex Laffer, Hilary Hicks, Genna Losinski, Amber Watts

**Affiliations:** University of Kansas, Lawrence, Kansas, United States

## Abstract

Lifestyle behaviors are important determinants of healthy brain aging. Research has not fully explored how sleep quality and physical activity may differentially influence specific domains of cognitive function. The present study aimed to estimate the relative influence of sleep quality and physical activity on cognitive performance in three domains in a sample of older adults. Older adults (ages 60-89, M = 74.74) without cognitive impairment (N= 160) wore an accelerometer for 7 days in a free-living environment. We used average vector magnitude counts per minute to measure total physical activity (TPA), and average wake after sleep onset (WASO) to measure sleep quality. We created cognitive composite scores (executive function, attention, and verbal memory) from neuropsychological data using confirmatory factor analysis. We regressed cognitive scores onto TPA and WASO with age and education entered as covariates. Higher amounts of physical activity and better sleep quality were associated with better executive function (R2 = 20.3%, F (4, 155) = 11.12, p < .001). Neither physical activity nor sleep quality was associated with verbal memory or attention. Results suggest that more physical activity and improved ability to stay asleep may benefit executive function, but not other cognitive domains. Future studies should clarify the interaction and mechanisms of action between health behaviors and cognitive performance in older adults.

